# Elevation Mechanisms and Diagnostic Consideration of Cardiac Troponins under Conditions Not Associated with Myocardial Infarction. Part 1

**DOI:** 10.3390/life11090914

**Published:** 2021-09-02

**Authors:** Aleksey M. Chaulin

**Affiliations:** 1 Department of Cardiology and Cardiovascular Surgery, Samara State Medical University, 443099 Samara, Russia; a.m.chaulin@samsmu.ru; Tel.: +7-(927)-770-25-87; 2 Department of Histology and Embryology, Samara State Medical University, 443099 Samara, Russia

**Keywords:** acute myocardial infarction, troponin T, troponin I, physical activity, myocarditis, endocarditis, sepsis, pulmonary embolism, chronic kidney disease

## Abstract

Although cardiac troponins are considered the most specific biomarkers for the diagnosis of acute myocardial infarction (AMI), their diagnostic consideration goes far beyond the detection of this dangerous disease. The mechanisms of cardiac troponin elevation are extremely numerous and not limited to ischemic necrosis of cardiac myocytes. Practitioners should be well aware of the underlying pathological and physiological conditions that can lead to elevated serum levels of cardiac troponins to avoid differential diagnostic errors, which will be greatly increased if clinicians rely on laboratory data alone. This article presents a classification of the main causes of an elevation in cardiac troponins and discusses in detail the mechanisms of such elevation and the diagnostic consideration of cardiac troponins in some conditions not associated with AMI, such as physical exertion, inflammatory heart diseases (myocarditis and endocarditis), pulmonary embolism (PE), renal failure, and systemic inflammation (sepsis).

## 1. Background

### 1.1. Classification of Causes Accompanied by an Elevation in the Concentration of Cardiac Troponin Isoforms in the Absence of Myocardial Infarction

The troponin complex is the most important regulator of the contraction/relaxation of striated muscle tissues (cardiac and skeletal) and consists of three troponin isoforms (subunits): troponin T, troponin I, and troponin C. Each of these proteins performs a specific function. Troponin T, or a tropomyosin-binding subunit, interacts with tropomyosin and attaches the troponin complex to actin filaments. Troponin I, or an inhibitory subunit, inhibits interaction of actin filaments with myosin heads in the absence of calcium ions in the cardiac diastolic phase. Troponin C, or a calcium-binding subunit, binds itself to calcium ions in the cardiac systole phase initiating conformational changes in the troponin complex to enable muscle contraction. The amino acid structure of troponin T and troponin I is different in cardiac and skeletal muscles, while the amino acid composition of troponin C is exactly the same in cardiac and skeletal muscles. Therefore, troponin C does not possess the required specificity to diagnose acute myocardial infarction (AMI) [1,2].

The cardiac isoforms of troponins T and I, due to their specificity, are the most common markers for the diagnosis of myocardial infarction [1,2,3]. An increase in cardiac troponins in biological fluids, in particular in blood, indicates reversible or irreversible damage to cardiomyocytes but does not explain the etiopathogenesis of this damage, peculiar for several other pathological processes not associated with myocardial infarction [1,2,4,5]. Moreover, in some cases (impaired renal filtration and false-positive results), cardiac troponin elevation can occur even in the absence of damage to cardiomyocytes [6,7,8,9].

Elucidation of all the causes and mechanisms of an increase in cardiac troponins has both theoretical and practical consideration and is necessary to improve the differential diagnosis of myocardial infarction from other conditions accompanied by non-ischemic alteration of cardiomyocytes [10,11,12].

**Figure 1:**
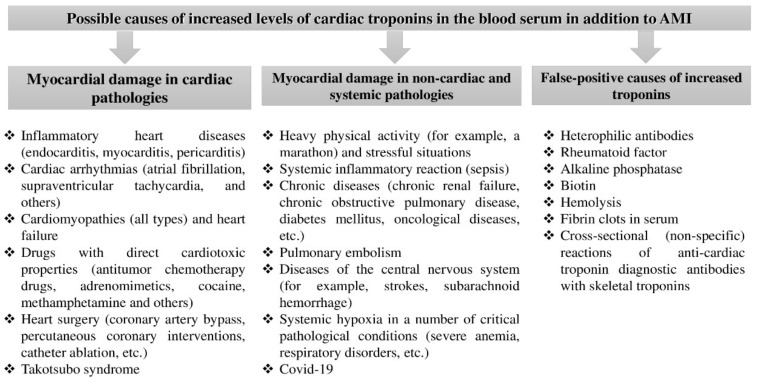
Classification of the main causes of elevated troponins not associated with myocardial infarction [4].

Based on the literature data, the most significant causes of elevation of cardiac troponins T and I, complicating differential diagnosis, include heavy and prolonged physical activity, inflammation of the heart wall layers (pericarditis, endocarditis, and myocarditis), cardiomyopathies, heart failure, pulmonary embolism (PE), systemic inflammation (sepsis), acute and chronic kidney disease, cardiotoxicity of some therapeutic agents and heart surgeries, and skeletal muscle damage [1,4,12] (Figure 1). False-positive factors (interferences) that contribute to the overestimation of troponin levels are considered equally important problems in laboratory diagnostics [5,6,7,8,9].

Notable that improving the sensitivity of troponin immunoassays not only has positive aspects (accelerating diagnosis and improving the prognosis of patients) [1] but also leads to a decrease in specificity, which is expressed by more frequent cases of elevated troponins in the absence of myocardial infarction [1,4,13,14]. In addition, highly sensitive detection methods register much more cases of increased troponins in a series of other pathological processes (myocarditis, sepsis, pulmonary embolism, etc.) that damage the myocardium. On the one hand, this can have an important prognostic value, presuming more adequate therapy, and on the other hand, it further complicates differential diagnosis and contributes to misdiagnosis or overdiagnosis. This is especially true in cases where clinicians rely primarily upon laboratory data [11,12,13,14,15,16,17].

Medical institutions in many countries are gradually switching to the use of highly and ultrasensitive troponin test systems. Anand A. et al. conducted a large study to estimate the global prevalence of highly sensitive assays. The authors, using a questionnaire, collected information from 1902 institutions distributed in 23 countries all over the world. In total, 96% of medical centers used cardiac troponins as the main biomarkers of myocardial infarction, of which 41% employed highly sensitive analyses and an accelerated algorithm for diagnosing myocardial infarction (0 → 3 h) [18].

An official document, the fourth universal definition of myocardial infarction (UDMI) from 2018, regulates the use of highly sensitive immunoassays with accelerated diagnostic algorithms for the early detection of myocardial infarction: the determination of troponin concentration immediately upon admission and then within 1–3 h (0 → 1 h, 0 → 3 h). Moreover, an earlier diagnosis contributes to the early onset of correct therapy and a significant improvement in the prognosis of admitted patients [1].

The guidelines of the European Society of Cardiology (ESC) emphasize the high efficiency of using early algorithms (0 → 1 h and 0 → 2 h) in order to exclude or confirm myocardial infarction without ST-segment elevation (NSTEMI) [19]. These early NSTEMI diagnostic algorithms are based on estimating the levels of high-sensitivity cardiac troponins at the time of patient admission and then the repeated estimation of concentration levels after 1 or 2 h. The threshold levels of high-sensitivity cardiac troponins in order to confirm/exclude NSTEMI are specific for immunoassays by different manufacturers (Table 1).

If the level of high-sensitivity cardiac troponins measured at admission is very low or low and there is no significant increase thereof in 1 (or 2) hours, then NSTEMI diagnosis is excluded, and such patients are recommended for early release and ambulant or elective treatment. If the level of high-sensitivity cardiac troponins measured at admission is above the thresholds or upon repeated estimation in 1 (or 2) hours there is a significant increase thereof, then the probability to diagnose NSTEMI is high, and such patients are recommended to be hospitalized. The timing of an invasive strategy is secondary to risk stratification [19].

However, it is important to realize that the increase in cardiac troponin level is not always a sign of AMI (’ischemic necrosis of cardiomyocyte’) but can be indicative of any type of damage to the cardiomyocytes (except for the cases of false-positive elevation) and can be observed in many, not related to AMI, physiological (e.g., physical exertion and psycho-emotional stresses) and pathological conditions (Figure 1). This research paper consistently discusses the causes and mechanisms of cardiac troponin elevation under physical activity, inflammatory myocardial diseases, pulmonary embolism, chronic renal failure, and sepsis.

### 1.2. Causes and Mechanisms of Elevated Cardiac Troponins during Physical Activity

Physical activity is one of the widespread reasons for an increase in the concentration of cardiac troponins in the blood serum. Rifai N. et al. were among the first to study cardiac troponins T and I (cTnT and cTnI) in athletes (n = 23) participating in the Ironman triathlon in Hawaii (swimming 3.9 km → cycling 180.2 km → running 42.2 km). The athletes’ concentrations of troponins were measured several days before the start and immediately after the finish, parallel with electrocardiography (ECG) and echocardiography (EchoCG). After the triathlon, increased troponin values were recorded in 26% of the athletes. The highest concentrations of cTnT were observed in athletes who finished in prize-winning places. Moreover, there was a significant correlation between finish and cTnT values (r = −0.65; *p* < 0.01). According to EchoCG data, the ejection fraction of athletes after the finish decreased by an average of 24% in comparison with the indicators before the start (*p* < 0.002). The results obtained prompted the suggestion that such a heavy load is undesirable, as it damages the myocardium [20]. Mingels A. et al. determined the concentration of cardiac troponins in 85 marathon runners using moderately sensitive (cTnT and cTnI) and highly sensitive immunoassays (hs-cTnT) before the start, after the finish, and 24 h after the race. Immediately after the finish, the hs-cTnT level was increased in 86% of the marathon participants, and cTnT and cTnI increased in 45 and 81% of the runners, respectively. Serum concentrations of cardiac troponins increased approximately 10-fold. There was a close and significant correlation between the concentrations of hs-cTnT and cTnT at the finish line (r = 0.955; *p* < 0.001). Multiple regression analysis showed that higher levels of cardiac troponins were found in older runners with less athletic experience. In 24 h after the race, there was a decrease in troponin levels, but they were still 3–4 times higher than the initial concentrations [21].

Scherr J. et al. studied the longer term kinetics of many inflammatory, cardiac biomarkers, including hs-cTnT, in marathon runners (n = 102). The authors measured the concentration before the start, immediately after the finish, then 24 and 72 h after the race. The concentration of hs-cTnT after the marathon increased by about 10 times in comparison with the indicators before the start (from 0.00361 ng/mL to 0.031 ng/mL), and the complete normalization of hs-cTnT levels was reached 72 h after the finish [22]. A recent study by Richardson A.J. et al. also revealed a significant rise in hs-cTnT levels (more than 10 times) in 52 marathon runners in Brighton (UK). The hs-cTnT levels measured two days before the race and 10 after minutes were 5.60 ± 3.27 ng/L and 74.52 ± 30.39 ng/L, respectively (*p* < 0.001). The researchers also noted that the magnitude of the increase in hs-cTnT is associated with the intensity of physical activity against the respiratory threshold, heart rate (HR), and maximum oxygen consumption [23].

Lazzarino A. et al. first noted the relationship between neurosis (the concentration of cortisol) and increased levels of troponin T measured by a high-sensitivity test system (RocheDiagnostics). The authors examined 508 persons without signs of coronary artery disease. According to scientists, the further establishment of the exact role of stress in the pathophysiology of cardiomyocyte damage is an urgent direction [24].

Discussions are ongoing regarding the mechanisms of troponin elevation during physical activity. Some researchers believe that prolonged and heavy loads cause subclinical (small-scale) necrosis of contractile cardiomyocytes, and more pronounced hypertrophy of the remaining cardiomyocytes (compensation), further increasing the risk of cardiosclerosis (decompensation), which is clinically manifested by heart failure. Perhaps the frequency and intensity of exercise are associated with the risk of heart failure in athletes. Most researchers think that heavy loads lead to transient myocardial ischemia and reversible damage to cardiomyocytes [25,26,27,28]. One of the arguments in favor of this statement is kinetic changes in troponin levels. Thus, in myocardial infarction, the duration of the circulation of troponin proteins is 1–2 weeks, whereas, even with excessive physical exertion, it is no more than 1–3 days [25,26]. The second argument supporting the reversibility of myocardial damage is the data of magnetic resonance imaging (MRI) with gadolinium preparations contrast. This method, following the modern development of science, is ideal for visualizing inflammation and myocardial fibrosis. O’Hanlon R. et al. visualized myocardial tissue in 17 athletes (11 of them had elevated cTnI levels) by MRI and found no signs of necrosclerotic changes [27]. Based on the research results, Paana T. et al. concluded that cardiac troponin elevation after a marathon race is a common and non-hazardous phenomenon and is not associated with the markers of coronary atherosclerosis, vulnerability for plaques, or markers of skeletal muscle injury [28]. When studying the effect of intensive and long physical activity on the rate of troponins release from cardiomyocytes, it was proved that intensity plays a more significant role. At that, physical activities of low intensity did not cause any reliable increase in the levels of troponin I, and after physical activities of high intensity, the level of troponin I was significantly higher than after physical activities of moderate intensity [29]. Moreover, among the additional factors influencing the release of troponins from the myocardium in the course of physical activity are the duration of heart rate increase during physical activities [30] and the presence of latent obstructive lesion of coronary arteries in people engaged in sports [31].

Transient ischemia during prolonged or heavy physical exertion is probably due to an imbalance between the demand for oxygen in cardiomyocytes, substrates for the energy supply of the myocardium, and their delivery through the coronary arteries. Myocardial tissue is known to be very sensitive to hypo-oxygenation due to the low reserves of myoglobin, glycogen, and other energy resources. Therefore, against the background of increased demand for the myocardium, mediated by tachycardia, the blood supply to the myocardium is reduced due to vasospasm. The main part here belongs to catecholamines and cortisol, which lead to the temporary disruption of myocardial metabolism and reversible damage to cardiomyocytes [32,33,34].

It is believed that one of the mechanisms for the release of troponins through the intact membrane of a cardiomyocyte is blebbing vesicles (membrane vesicles) with cardiomyocyte proteins inside them. Schwartz P. et al. for the first time observed membrane vesicles in an in vitro experiment using electron microscopy. In this case, the number of blebbing vesicles on the surface of the cardiomyocyte increases respectively to the duration of ischemia, and when it is eliminated, the vesicles are reabsorbed back into the cytoplasm. However, there is still no reliable evidence in favor of the functioning of this mechanism in vivo [34].

Stress echocardiography with mechanical stress (exercise, bicycle ergometer, etc.) or drugs (dobutamine, dipyridamole, etc.), as shown by the recommendations [35,36], is considered the most important tool in the diagnosis coronary atherosclerosis and allows the monitoring and predicting of the unfavorable course of ischemic heart disease (IHD). Nevertheless, the methods of functional diagnostics, undoubtedly, are less sensitive than methods of laboratory diagnostics.

Samaha E. et al. in a prospective study studied the kinetics of hs-cTnT concentration (Roche Diagnostics Elecsys 2010) in patients (n = 48) after stress echocardiography. Thirty-three patients underwent stress echocardiography with exercise, and fifteen patients underwent stress echocardiography with pharmacological (dobutamine) stress. Hs-cTnT levels were measured before and 30 min, 1–2, and 4–6 h after completion of stress echocardiography. The maximum increase in hs-cTnT concentration was observed 4–6 h after stress echocardiography. At the same time, the increase in hs-cTnT concentrations was higher after stress echocardiography with dobutamine (n = 15; median ∆hs-cTnT + 9.7 ng/L) than after stress echocardiography with exercise (n = 33; median ∆hs-cTnT + 2.3 ng/L). However, these researchers found no association between elevated troponin levels and stress-induced myocardial ischemia as measured by echocardiography [37].

The studies that showed a direct relationship between the stage of IHD and elevated levels of cardiac troponins in the serum and oral fluid [38,39], as well as the presence of a significant correlation between hs-cTnT and the intensity of physical activity [23], suggests that the determination of troponins using high-sensitivity techniques can more accurately and reliably detect concealed myocardial ischemia than stress echocardiography and stress ECG or become an important additional criterion for the diagnosis and stratification of IHD after exercise tests. However, the works in this area are still scarce and include a relatively small number of patients, and further study using larger samples is required for clarification.

Manjunath L. described a case of overestimated troponin I (0.123 ng/mL at a rate of <0.055 ng/mL) in a young patient who was admitted to the emergency department with chest discomfort. Doctors suspected myocardial infarction, additionally supported by unfavorable family history and the presence of hypercholesterolemia. However, ECG, Echo-KG, and coronary angiography did not reveal any signs of myocardial ischemia. The studied anamnesis clarified that the young man was actively involved in sports, and the day before admission, he ran several miles preparing for a marathon [40].

Thus, the elevation of the levels of cardiac troponins is a common effect during heavy physical exertion, while highly sensitive methods register the troponin level diagnostic thresholds crossing much more often than moderately sensitive ones. Such conditions may pose issues in differential diagnosis, but a thorough history taking (a physical activity the day before admission), as well as taking into account the dynamics of concentration (faster decrease), and the use of additional methods, allow avoiding overdiagnosis and subsequent unnecessary treatment and hospitalization costs. It is also noted that the use of highly sensitive methods for the determination of troponins has great prospects in the diagnosis and prediction of IHD concealed forms using stress tests.

### 1.3. Causes and Mechanisms of Elevated Cardiac Troponins in Inflammatory Heart Diseases (Endo-, Peri-, and Myo-Carditis)

An increase in cardiomarkers in myocarditis is due to the direct damaging (cytotoxic) effect of infectious agents (viruses, bacteria, etc.), toxins, and autoantibodies on cardiomyocytes. At the same time, troponin concentrations vary widely. Thus, in several studies, troponins, determined by moderately sensitive methods for the diagnosis of myocarditis, had a sensitivity of 34–71% and a specificity of 86–94% [41,42,43,44,45].

High-sensitivity troponins are much more likely to increase in myocarditis and have certain advantages over other methods and biomarkers. Thus, Ukena C. and colleagues, for the first time, studied the concentration of hs-cTnT, copeptin, and the N-terminal precursor of natriuretic hormone (NT-proBNP) in patients with suspected myocarditis (n = 70). According to endomyocardial biopsy, which is considered the “gold standard” in detecting myocarditis, patients were conditionally divided into three groups: (1) acute myocarditis was diagnosed in 6 patients, (2) chronic myocarditis in 36, and (3) absence of inflammation in 28 patients. In acute myocarditis, the highest concentrations of hs-cTnT were observed (262.9 pg/mL (61.4–884.2)), compared with patients with chronic myocarditis (20.4 pg/mL (15.6–20.4); *p* < 0.0001), and with patients with no inflammation (19.5 pg/mL (13.8–50.7); *p* < 0.0001). The concentration of hs-cTnT ≥ 50 pg/mL had high sensitivity and specificity in the diagnosis of acute myocarditis. At the same time, the levels of copeptin and NT-proBNP did not significantly differ between these groups and, accordingly, were ineffective in the diagnosis of myocarditis [46].

According to accumulated information, endomyocardial biopsy has shortcomings in the detection of viral myocarditis since it does not reflect the presence of the viral genome in cardiomyocytes and, accordingly, will not “see” inflammation in the case of a weak course of chronic viral myocarditis [45]. The pathogenetic significance of viral persistence in the myocardium is not fully understood. Ukena C. et al. using polymerase chain reaction investigated the viral genome (enteroviruses (Coxsackie), parvovirus B19, adenovirus, herpes viruses (Epstein–Barr)) in patients with suspected myocarditis. It is noteworthy that the levels of hs-cTnT were significantly higher (*p* = 0.042) in patients with the detected viral genome (37.4 pg/mL (21.9–163.6)) compared with those patients in whom viral nucleic acid particles were absent (20 pg/mL (14–44.4)) [46]. This indicates the damaging effect of viruses on cardiomyocytes, which cannot be detected by endomyocardial biopsy, especially with weak process activity. Thus, the use of hs-cTnT in myocarditis has a significant advantage over endomyocardial biopsy and moderately sensitive methods of detecting cardiac troponins in the detection of viral myocarditis.

Due to the high mortality rate of newborn infants and children from myocarditis, averaging 75 and 25%, respectively, a timely diagnosis and severity-adequate therapy are of paramount importance [47]. Since the clinical picture of childhood myocarditis in many cases can proceed under the guise of other diseases, most often influenza, or even be asymptomatic, many researchers prioritize the search for biomarkers for an early diagnosis and the determination of an unfavorable prognosis.

According to Soongswang J. et al., the concentration of cTnT > 0.052 ng/ml has a sensitivity of 71% and a specificity of 86% for the diagnosis of myocarditis in children [42,43]. A recent large retrospective review of medical records over a 12-year period in a pediatric hospital found that deaths were much more common in children with elevated levels of cardiac troponin I, a creatine kinase-MB isoform. At the same time, increased concentrations of these biomarkers were associated with arrhythmia, hypotension, acidosis and decreased left ventricular ejection fraction, and gastrointestinal symptoms. The vast majority of patients died within the first 72 h. In a multivariate analysis, very high cTnI levels (>45 ng/mL) and decreased left ventricular ejection fraction (<42%) were associated with a high risk of death, especially in the first 24 h. According to the authors, more intensive therapy is justified for individuals with high cTnI concentrations [47,48].

Abrar S. et al. conducted an observational study of pediatric patients with suspected myocarditis. The cTnI concentrations and echocardiographic data contributed to the correct diagnosis, with the mean cTnI levels (0.958 ± 1.13 ng/mL) being higher in the group of non-surviving patients (*p* = 0.0074). Apart from troponin I, other factors that predisposed to poor outcomes were hypotension, natriuretic peptide (BNP) levels, and decreased ejection fraction [49].

However, in some cases, myocarditis is not accompanied by an increase in the concentration of cardiac troponins. This raises the question: why are troponins, which have a high sensitivity (about 90–100%) in ischemic myocardial damage, not such effective markers for the diagnosis of myocarditis? This is likely due to the diagnostic characteristics (primarily sensitivity) of the test systems used. Beyond that, it is assumed that this is due to analytical interferences arising from the presence of autoantibodies to cardiac troponins in the blood serum of patients with myocarditis. Japanese researchers Matsumori A. et al. [50] revealed a significant increase in the titer of autoantibodies to troponin I in patients with myocarditis. It is possible that these autoantibodies, binding the epitopes of circulating troponin molecules, reduce their availability for commercial antibodies in the diagnostic kit. Experiments on animal models have also shown that autoantibodies to troponins are involved in the pathogenesis of myocarditis and cardiomyopathies. The interaction of troponin autoantibodies with myocardial cells increases the intracellular content of calcium ions, cardiac dysfunction, dilatation of the heart chambers, and the occurrence of heart failure [50].

Although endocardial and pericardial cells do not contain cardiac troponins, endocarditis and pericarditis are often accompanied by elevated troponin levels. This is likely due to the involvement of myocardial tissue in the inflammatory process, because of the tight location of the heart wall layers. Researchers recorded an increase in cTnI concentration in 11 out of 15 (73%) patients with confirmed infective endocarditis. None of the troponin-negative patients died, while two troponin-positive patients passed, indicating a worse prognosis for patients with high cTnI. At the same time, there was no correlation between elevated cTnI concentrations and the need for valve replacement in these patients. The authors also noted that all patients with staphylococcal endocarditis had elevated cTnI levels, while among those with streptococcal endocarditis, only 50% were marked with such elevation [51]. It is possible that the etiology of endocarditis can be associated with increased concentrations of cTnI and, accordingly, can indicate a greater aggressiveness of some infectious agents to the myocardium, but further larger studies are needed to establish this.

Tsenovoy R. and colleagues studied a larger number of patients with infective endocarditis: an increase in troponin levels above the reference (>0.4 ng/mL) was observed in 35 out of 62 people (57%). It is noteworthy that among troponin-positive patients, in-hospital mortality or the necessity for valve replacement surgery occurred more frequently (51 versus 15%, *p* < 0.005). As a result of hemoculture, Staphylococcus aureus was detected in 71% of troponin-positive individuals [52].

Purcell J.B. et al. in a prospective study concluded that elevated cardiac troponin I levels in patients with myocarditis are associated with a combination of adverse events, such as death, myocardial abscess, and central nervous system damage (*p* < 0.001) [53].

In a large study that included 118 patients with viral or idiopathic viral pericarditis, cTnI concentrations exceeded threshold values (>1.5 ng/mL) in 38 people (32.2%). Positive troponin I was significantly associated with younger age (*p* < 0.001), male sex (*p* = 0.007), ST-segment elevation (*p* < 0.001), and pericardial effusion (*p* = 0.007). At the same time, cTnI was not a negative prognostic marker [54].

In another study by Gamaza-Chulian S. et al., an increase in cardiac troponin T was recorded in 64 of 105 patients with pericarditis (60.9%). Only young age was associated with higher cTnT levels in a multivariate analysis (*p* = 0.03). Fatal cases were not recorded in the hospital, which indicates that cTnT is not a negative prognostic marker of acute pericarditis [55].

Thus, the levels of cardiac troponins often increase in inflammatory lesions of the heart membranes, which leads to errors in the differential diagnosis. In cases of endocarditis and myocarditis, apart from pericarditis, elevated cTnT and cTnI may be considered prognostic markers. Wide ranges of concentrations in these nosologies can be associated with different sensitivity of immunoassays, and in cases with myocarditis, the additional action of autoantibodies against cardiac troponins can lead to analytical errors (interferences).

### 1.4. Diagnostic Valuae and Mechanisms of Elevation in Cardiac Troponins in PE

Cardiac troponins, according to the clinical guidelines of the European Society of Cardiology (ESC) for the diagnosis and treatment of acute pulmonary embolism, are one of the main biomarkers for predicting and stratifying the risk of pulmonary embolism [56,57,58].

According to Giannitis E. et al., an increase in cardiac troponins depends on the severity of the thrombotic occlusion of the vascular bed of the lungs: with massive PE on average in 50% of patients; with submassive in about 35%; and with non-massive PE, it practically does not occur. Inpatient mortality, cardiogenic shock, and the need for resuscitation were more common in patients with elevated cTnT values. Troponin-positive patients more often required inotropic support and mechanical ventilation (MV) [59].

Turkish researchers led by Kilinc G. determined the concentration of cTnI using a moderately sensitive immunoassay (Beckman Coulter). Of 106 patients admitted to the hospital with suspected PE, the diagnosis was confirmed in 63 people. At the same time, increased cTnI levels were found much more often in patients with confirmed PE compared with patients in whom the diagnosis of PE was not confirmed: 50.8% (n = 32) versus 11.6% (n = 5); *p* < 0.001, respectively. High levels of cTnI were recorded in 80% of patients with massive PE, 56.25% with submassive PE, and 38.4% with non-massive pulmonary embolism. It is also calculated that a high level of cTnI in the diagnosis of pulmonary embolism has a sensitivity of 50.8% and a specificity of 88.3%. When analyzing a combination of elevated levels of cTnI and D-dimer, the sensitivity in the diagnosis of PE increased to 93.5%, but the specificity decreased to 54.5% [60].

Becattini S. et al. performed the most detailed meta-analysis on the predictive value of moderately sensitive troponins in PE. The analysis included 20 studies (1985 patients). The predictive value of cTnI and cTnT was approximately the same. Among 618 patients with elevated levels of cardiac troponins, 122 died ((19.7%; 95% confidence interval [CI] 16.6–22.8)). In contrast, among 1367 subjects with reference troponin values, the deaths were much less frequent—51 (3.7%; 95% CI 2.7–4.7). High troponin concentrations are significantly associated with short-term patient mortality (odds ratio (OR) 5.24; 95% CI 3.28–8.38). In the subgroup of hemodynamically stable patients, elevated troponins were also associated with high mortality (OR 5.90; 95% CI 2.68–12.95). Taking this information into account, the authors made an important conclusion: increased concentrations of cardiac troponins in PE allow the identification of patients with a high risk of short-term death and unfavorable outcomes [61].

The kinetics of cTnT concentration in PE and ACS is different, which may indicate different mechanisms of troponin release. In a study, 56% of patients with acute PE were cTnT positive on admission. After 10 h, cTnT concentrations reached peak values (median 0.48 μg/L), and the duration of circulation at the increased concentrations was 30–40 h. In myocardial microinfarction, the peak values were in the range of 0.22–0.41 μg/L, the concentration curves had several repetitive up and down slopes, and the duration of cTnT circulation was more than 120 h [62].

Lankeit M. et al. prospectively investigated the predictive value of moderately and highly sensitive troponin T in 156 patients with a confirmed diagnosis of PE. In 64% of patients (n = 100), hs-cTnT values exceeded the 99th percentile threshold (>14 ng/mL). Patients with elevated hs-cTnT concentrations had a reduced likelihood of long-term survival (*p* = 0.029 for the log-rank test). Moreover, according to Cox regression, hs-cTnT was the only biomarker predicting an increased risk of death in the long term. In turn, approximately 50% of patients with poor prognosis had normal levels of moderately sensitive cTnT and, accordingly, could be mistakenly included in the group of patients with a favorable prognosis. This study demonstrates a significant advantage of hs-cTnT over the conventional cTnT test for assessing the prognosis of patients with PE [63].

One of the most important indicators that determine the prognosis and severity of PE is right ventricular dysfunction. The likelihood of death in the case of right ventricular dysfunction is 2–5 times higher than in patients without it. Cardiac troponins are associated with the presence and degree of right ventricular dysfunction and may be used as an alternative or adjunct to echocardiographic PE assessment. This also indicates that troponin molecules are released from right ventricular cardiomyocytes as a result of their death or reversible damage, which explains the importance of determining the concentration of cardiac troponins in PE [64]. According to Cotugno M. et al., the concentrations of cTnT and NT-proBNP in patients with right ventricular dysfunction in acute PE were significantly higher than in those without it. Moreover, NT-proBNP had a higher sensitivity in detecting right ventricular dysfunction than cTnT [65]. Establishing a short-term and long-term prognosis is important for the treatment and diagnostic process. Thus, patients with a good prognosis (low troponin levels) can receive less aggressive treatment and stay in the hospital for a shorter time. Troponins, as measured by highly sensitive immunoassays, are significantly better suited for patient stratification than moderately sensitive methods. However, it should be understood that the use of highly sensitive analyses can complicate the differential diagnosis of PE and acute myocardial infarction.

To solve the problem of differential diagnosis, Kim J.Y. et al. used the D-dimer/troponin I ratio. The authors conducted a retrospective study that included 771 patients with myocardial infarction and 233 patients with acute PE. An increase in the levels of D-dimers was recorded in about half (49.5%) of patients with myocardial infarction, and the concentration of cTnI was increased in 38.6% of patients with PE. The threshold values for differentiating PE and myocardial infarction made 1.12 mg/L for D-dimer (sensitivity 81.1%, specificity 70.2%) and 0.72 ng/mL for cTnI (sensitivity 80.6%, specificity 78.9%). Moreover, when using the value of the ratio D-dimers/cTnI > 1.82 in the differential diagnosis of PE from myocardial infarction, the sensitivity and specificity were significantly higher than the diagnostic value of individual markers and amounted to 93.3 and 86.6%, respectively. The authors believe that by using this ratio, an invasive coronary angiography procedure can be avoided [66].

The mechanisms of increasing cardiac troponins in non-massive PE are most likely associated with the release of the cytosolic pool through the membrane of the right ventricular cardiomyocytes, due to increased afterload, which is similar to how it occurs during intense physical exertion [67,68,69]. In the case of submassive and massive PE, necrosis of cardiomyocytes happens. It occurs due to a sharp overload and expansion of the right ventricle, which leads to compression of the small branches of the coronary arteries passing through the myocardium, and impaired hemoperfusion [62,63]. This is confirmed by pathological studies that revealed necrotic foci in the myocardium of the right ventricle with intact coronary arteries [64,70,71].

### 1.5. Causes and Mechanisms of Elevated Cardiac Troponins in Chronic Kidney Disease

Following modern concepts, when using the latest (highly sensitive and ultrasensitive) immunoassays, cardiac troponins can be considered as products of normal myocardial metabolism (“there are no more troponin-negative patients”) [25,72,73,74], since they are found in all healthy patients in a concentration less than the threshold level (99th percentile).

However, it is also important to understand that the concentration of cardiac troponin isoforms in the blood serum depends not only on the mechanisms of troponin release from cardiomyocytes but also on the mechanisms of their elimination from the bloodstream. The most well-known methods for removing many proteins, in particular troponins, include cleavage by specific proteases inside the cells of the reticuloendothelial system; extracellular cleavage by proteases, such as thrombin; and renal filtration [75,76,77,78,79,80].

The most controversial mechanism for the elimination of troponins is glomerular filtration. Some researchers denied this mechanism of troponin removal since it was impossible to determine troponins in urine [78]. An indirect confirmation of the involvement of the kidneys in the elimination of troponins from the blood was the reports of clinicians about overestimated levels of troponins in patients suffering from chronic kidney disease without coronary artery disease. The most striking research confirming this argument was presented by Dubin R.F. et al., who studied 2464 participants in a multinational cohort of chronic kidney disease (CRIC study). At the same time, hs-cTnT was increased in 81% of patients with chronic kidney disease but without obvious signs of cardiovascular diseases. In patients with more severe CKD, hs-cTnT concentrations were higher: patients with a glomerular filtration rate (GFR) of <30 mL/min, on average, had three times higher hs-cTnT values compared with patients whose GFR was >60 mL/min [81].

The work of Croatian researchers led by Pervan P. provides direct evidence for the involvement of the kidneys in the processes of troponin I elimination. Researchers found hs-cTnI by a highly sensitive method (Abbott Architect i1000SR) in all examined patients: in the collected morning urine of normotensive patients, hs-cTnI concentrations were 14.95 pg/mL compared to urinary levels of hs-cTnI = 26.59 pg/mL in patients with high blood pressure (*p* < 0.05). The authors believe that the determination of the level of hs-cTnI in urine can be used in the diagnosis and monitoring of hypertension [77]. Given that blood pressure increases GFR in direct proportion, this study also provides strong evidence for a GFR relationship for serum troponin levels. Thus, in patients with higher GFR mediated by hypertension, the concentration of troponin in the urine is correspondingly higher. GFR is currently calculated primarily based on the endogenous metabolite creatinine. A study by Wilhelm J. et al. found a significant correlation between serum creatinine and hs-cTnT levels (r = 0.554; *p* < 0.001) [82]. In a study by Rosjo N. et al., the correlation between serum concentrations of hs-cTnT and creatinine was r = 0.32, *p* < 0.001 [83]. These data prove the relationship between elevated levels of cardiac troponins and impaired renal elimination (decreased GFR).

Nevertheless, not all researchers succeeded in confirming the relationship between the fall in GFR and the increased concentrations of cardiac troponins, as well as detecting troponin in urine [84,85]. It is assumed that this is primarily due to the analytical capabilities of the immunoassays used: highly sensitive and ultrasensitive assays have a much stronger detecting ability and can find troponins where conventional (moderately sensitive) test systems fail.

Two more hypotheses intend to explain the increase in cardiac troponins in chronic kidney disease: (1) the “skeletal hypothesis” [86,87] and (2) the direct damaging effect on cardiomyocytes of toxins accumulated in chronic kidney disease [88,89].

The skeletal hypothesis is based on reports of scientists on the expression of cardiac troponin isoforms in skeletal muscle. In chronic renal failure, skeletal muscle alteration (uremic skeletal myopathy) occurs, followed by reparative regeneration processes, during which, as some scientists believe, the expression of cardiac troponin isoforms occurs. Ricchiutti V. et al. reported the detection of messenger RNA of cardiac troponin T in skeletal muscle biopsies in 50% of patients on hemodialysis due to chronic kidney disease [86]. Haller C. and colleagues did not find cardiac troponin T isoforms in biopsies of the skeletal muscles of the abdominal wall in five patients with end-stage CKD [87]. Thus, the data on the expression of cardiac isoforms in skeletal muscles in uremic myopathy are controversial.

A long course of chronic kidney disease leads to overload and gradually increasing compensatory myocardial hypertrophy due to an increase in the volume of circulating blood. In conditions of sharp hypertrophy, narrowing of the subepicardial branches of the coronary arteries may happen as a result of their compression, which leads to the release of troponins due to the death of cardiomyocytes and the development of cardiosclerosis. It proves the fact that in some people who died from chronic kidney disease, postmortem examination revealed foci of microinfarction and cardiosclerosis [88,90]. Thus, the main mechanism of cardiac troponin elevation in chronic kidney disease has not been finally established. The most likely mechanism is decreased renal elimination. However, any other mechanisms should not be ruled out. Because of the relative paucity of fundamental research and their certain inconsistency, there is a need for further study and clarification.

### 1.6. Diagnostic Value and Mechanisms of Elevated Cardiac Troponins in Sepsis

The mechanisms of increasing cardiac troponins in sepsis are diverse. One of them is myocardial ischemia, which occurs due to an imbalance between the need for oxygen in cardiomyocytes and its delivery. The imbalance between the demand and the need for myocardial oxygen is due to a variety of pathophysiological pathways. The most significant of them are fever, hypotension, respiratory failure (respiratory hypoxia), and impaired acid-base and water-electrolyte balances, as well as microcirculation disorders, which lead to a decrease in the hemoperfusion of all organs, including the myocardium. Therefore, against the background of fever and hypotension, tachycardia increases in conditions in which cardiomyocytes consume oxygen faster, but its delivery through the coronary arteries decreases. Acid-base disorders, in turn, are accompanied by a disruption in the work of enzyme proteins that provide energy processes in the myocardium (Krebs cycle, oxidation of fatty acids, etc.). The gradually forced (due to lack of oxygen) transition of the myocardium to anaerobic glycolysis leads to the additional production of lactate, the progression of acidosis, general hypoxia, and metabolic disorders, thereby closing the vicious pathogenetic circle. Under such conditions, reversible or irreversible damage (death) of myocardial cells leads to the release of troponins [24,91,92,93].

Some researchers believe that the main role in damage to cardiomyocytes in sepsis is played by inflammatory mediators, such as tumor necrosis factor-alpha (TNF-ɑ), interleukins (IL-1, IL-6), and bacterial exo- and endo-toxins, which have a direct cytotoxic effect on cardiomyocytes [92,93,94]. Kumar A. et al. in an experimental study showed that serum from patients with sepsis containing inflammatory mediators reduces the amplitude and rate of contraction of cardiac myocytes [95].

Additional reasons for the increase in cardiac troponins in patients with sepsis may include microthrombosis of myocardial vessels and increased apoptosis of cardiomyocytes [96,97,98]. In addition, it should be borne in mind that severe sepsis and septic shock are often accompanied by multisystemic disorders, including renal failure, in which the elimination of troponins from the blood is reduced [17,99].

Thus, the mechanisms of increasing cardiac troponins in sepsis can be diverse; their complex participation is very likely with the predominance of one of them in a particular situation.

A large meta-analysis by Bessiere F. et al., combining 13 original works with 1227 patients with sepsis, found that an increased level of troponin was significantly associated with an increased risk of death (OR 1.91; CI 1.62–2.24). The prevalence of elevated cardiac troponin concentrations in patients with sepsis was 61% (95% CI 58–64%) [100].

Wilhelm J. et al. determined the concentration of hs-TnT in patients (n = 313) admitted to the emergency department with sepsis. Elevated hs-TnT were found in 197 patients (62.9%). Higher hs-TnT values were recorded in patients with severe sepsis (52.6 pg/mL) and septic shock (65.1 pg/mL) compared with uncomplicated sepsis (14.5 pg/mL; *p* < 0.001). Nevertheless, despite significantly lower hs-cTnT concentrations, it was higher than the 99th percentile in 51.6% of patients with uncomplicated sepsis and 34.5% of patients without renal insufficiency. Moreover, this also indicates the contribution of additional mechanisms (in addition to impaired renal elimination) in the increase in the levels of cardiac troponins in sepsis. The concentration of troponin hs-cTnT correlated with inflammatory markers (IL-6 (r = 0.193; *p* < 0.005) and procalcitonin (r = 0.265; *p* < 0.001)), serum creatinine (r = 0.554; *p* < 0.001), and patient severity scale APACHE II (r = 0.654; *p* < 0.001). Hs-cTnT showed good predictive value and means; deceased patients had higher hs-cTnT levels than survivors (AUC 0.72; *p* < 0.001) [82].

Rosjo N. et al. investigated the concentration of cTnT and hs-cTnT in 207 patients with sepsis. Of these, 166 patients (80%) had hs-cTnT concentrations above the 99th percentile, while moderately sensitive troponin (cTnT) levels were elevated only in 86 people (42%). There was a correlation between hs-cTnT levels with the severity of the disease (on the SAPSII scale, r = 0.27, *p* < 0.001), multisystemic disorder (on the SOFA scale, r = 0.30, *p* < 0.001), and creatinine concentration (r = 0, 32, *p* < 0.001). The median hs-cTnT level was higher in the group of deceased patients than in the group of survivors (0.054 (0.022–0.227) versus 0.035 (0.015–0.111) μg/L, *p* = 0.047)), while moderately sensitive troponin T did not differ significantly in these groups (*p* = 0.14). The hs-cTnT concentrations in patients with septic shock were significantly higher than those in patients without shock (0.044 (0.024–0.171) versus 0.033 (0.012–0.103) μg/L; *p* = 0.03), while the levels of cTnT in patients with shock and without shock did not differ [83]. Based on the above, it can be concluded that hs-cTnT is much better suited for assessing the severity and survival of patients with sepsis than cTnT. However, due to such a frequent increase in hs-cTnT, difficulties in differential diagnosis may arise.

## 2. Conclusions

Analyses of the literature revealed that conditions such as intense physical activity, inflammatory heart disease (myocarditis, pericarditis, endocarditis), pulmonary embolism, sepsis, and renal failure are very common causes of elevated cardiac troponins. Modern highly sensitive analyses, despite their advantage over moderately sensitive analyses (earlier increase in blood in myocardial infarction), have a significant disadvantage (reduced specificity). This leads to a frequent increase in troponins in the above conditions, which should, by all means, be considered by clinicians when interpreting the results. High-sensitivity troponins are promising markers for detecting latent (concealed) coronary heart disease, especially in combination with physical and pharmacological stress tests. Cardiac troponins in sepsis, pulmonary embolism, endocarditis, and myocarditis can be used as prognostic markers, while highly sensitive analyses are much better suited for this role.

## Figures and Tables

**Table 1:** Threshold levels of high-sensitivity troponins used in ESC guidelines for some immunoassays produced by different manufacturers [19].

**Diagnostic Algorithm 0 → 1 h**					
Immunoassay, manufacturer	High-sensitivity troponin level, indicative of very low probability of NSTEMI, ng/L	High-sensitivity troponin level, indicative of low probability of NSTEMI, ng/L	Changes in high-sensitivity troponin levels in 1 h in order to exclude NSTEMI, ng/L	High-sensitivity troponin level, indicative of high probability of NSTEMI, ng/L	Changes in high-sensitivity troponin levels in 1 h in order to confirm NSTEMI, ng/L
hs-cTnT (Elecsys; Roche)	<5	<12	<3	≥52	≥5
hs-cTnI (Architect; Abbott)	<4	<5	<2	≥64	≥6
hs-cTnI (Centaur; Siemens)	<3	<6	<3	≥120	≥12
hs-cTnI (Access; Beckman Coulter)	<4	<5	<4	≥50	≥15
hs-cTnI (Clarity; Singulex)	<1	<2	<1	≥30	≥6
**Diagnostic Algorithm 0 → 2 h**					
Immunoassay, manufacturer	High-sensitivity troponin level, indicative of very low probability of NSTEMI, ng/L	High-sensitivity troponin level, indicative of low probability of NSTEMI, ng/L	Changes in high-sensitivity troponin levels in 2 h in order to exclude NSTEMI, ng/L	High-sensitivity troponin level, indicative of high probability of NSTEMI, ng/L	Changes in high-sensitivity troponin levels in 2 h in order to confirm NSTEMI, ng/L
hs-cTnT (Elecsys; Roche)	<5	<14	<4	≥52	≥10
hs-cTnI (Architect; Abbott)	<4	<6	<2	≥64	≥15
hs-cTnI (Centaur; Siemens)	<3	<8	<7	≥120	≥20
hs-cTnI (Access; Beckman Coulter)	<4	<5	<5	≥50	≥20
hs-cTnI (Clarity; Singulex)	<1	TBD	TBD	≥30	TBD

Abbreviations. hs-cTnT—high-sensitivity troponin T; hs-cTnI—high-sensitivity troponin I; TBD—to be determined.

## Data Availability

Not applicable.
